# Efficacy of lurasidone on anxiety symptoms in patients with schizophrenia: A pooled post hoc analysis of five randomized, placebo‐controlled trials

**DOI:** 10.1002/pcn5.70245

**Published:** 2025-11-18

**Authors:** Takahiro Nemoto, Miyuki Okumura, Hidenori Maruyama

**Affiliations:** ^1^ Department of Neuropsychiatry Toho University Faculty of Medicine Ota‐ku Tokyo Japan; ^2^ Department of Psychiatry and Implementation Science Toho University Faculty of Medicine Ota‐ku Tokyo Japan; ^3^ Regulatory Affairs Sumitomo Pharma Co., Ltd. Chuo‐ku Osaka Japan; ^4^ Medical Affairs Sumitomo Pharma Co., Ltd. Chuo‐ku Osaka Japan

**Keywords:** antipsychotic, anxiety, anxiolytics, lurasidone, schizophrenia

## Abstract

**Aim:**

Comorbid anxiety disorders and their symptoms are common in schizophrenia and may occur before a relapse. The aim of this post hoc analysis is to investigate the efficacy of lurasidone on comorbid anxiety symptoms in patients with schizophrenia.

**Methods:**

Data were pooled from five Phase 3 randomized, double‐blind, placebo‐controlled 6‐week trials of lurasidone treatment of schizophrenia, focusing on the 40 and 80 mg doses compared with placebo. Subgroup analyses were performed in two subgroups: severe (baseline Positive and Negative Syndrome Scale [PANSS] G2 anxiety score ≥ 4) and non‐severe anxiety (baseline PANSS G2 score < 4).

**Results:**

In the severe anxiety group, both the 40 and 80 mg doses of lurasidone significantly reduced PANSS G2 score from Weeks 2 to 6 compared to placebo. In patients not receiving concomitant anxiolytics, significant improvement in the PANSS G2 score was observed at Week 6 for lurasidone 40 mg compared to placebo. In both lurasidone dose groups, PANSS total and subscale scores were significantly reduced at Week 6 compared to placebo. For baseline‐to‐Week‐6 change scores, the PANSS G2 score and PANSS positive subscale score showed a moderate correlation. There were no new AEs of concern for lurasidone in this pooled analysis.

**Conclusion:**

The results suggest treatment with 40 or 80 mg/day of lurasidone improves anxiety symptoms in schizophrenia patients with moderate to severe anxiety, at least in part, through a direct effect, and this effect was demonstrated by lurasidone monotherapy, without the use of concomitant anxiolytics.

## INTRODUCTION

Clinically significant anxiety is frequently observed during the course of schizophrenia, from the prodromal stage to the chronic stage of illness, where it may occur before relapse.^1^, ^2^, ^3^ The prevalence rate of comorbid anxiety disorders in schizophrenia is almost one‐third; the most common anxiety disorder is social anxiety, followed by post‐traumatic stress.^2^ The presence of comorbid anxiety in schizophrenia is associated with an increase in suicide attempts,^4^, ^5^, ^6^ severity of psychiatric symptoms,^4^, ^7^ and decreased social function and quality of life.^8^, ^9^ Based on the prevalence and impact of anxiety symptoms in schizophrenia, it is considered important to treat comorbid anxiety disorders and anxiety symptoms adequately.

In treatment guidelines for anxiety disorders,^10^ benzodiazepines are not recommended as first‐line therapy but nonetheless are commonly used in clinical practice,^11^ including treatment for anxiety symptoms associated with schizophrenia,^12^ which is reported to be high in Japan.^13^ However, the evidence for the efficacy of benzodiazepines for the treatment of comorbid anxiety in schizophrenia is limited. In a meta‐analysis of six randomized clinical trials (RCTs) evaluating benzodiazepine augmentation of antipsychotics for those with schizophrenia, significant sedation effects were found for agitation only for the first 30 min of augmentation treatment (1 RCT, *N* = 45), and no augmentation effects were found for clinical response outcomes across the six trials whose periods were longer than 3 weeks (6 RCTs).^14^ In addition, there are concerns about the long‐term use of benzodiazepines in terms of abuse, dependence, and cognitive decline in the general population,^15^, ^16^ and increased mortality rates in patients with schizophrenia.^17^ Consequently, long‐term treatment with benzodiazepine is not recommended for patients with schizophrenia as well as for patients with other mental illnesses.

The evidence for the efficacy of atypical antipsychotics to treat anxiety symptoms in schizophrenia is relatively limited. Open‐label studies have reported efficacy for aripiprazole in comorbid social anxiety symptoms in patients with schizophrenia^18^ and for olanzapine in the treatment of anxiety symptoms.^19^ In addition, pooled post hoc analyses of placebo‐controlled clinical trials have reported that aripiprazole, olanzapine, quetiapine, and risperidone reduced the anxiety/depression factor score of the Positive and Negative Syndrome Scale (PANSS).^20^, ^21^, ^22^, ^23^, ^24^

Lurasidone is an atypical antipsychotic widely approved for the treatment of schizophrenia in countries around the world, including the United States and Japan. The pharmacological profile of lurasidone is that it acts as an antagonist to dopamine D_2_, serotonin 5‐HT_2A_ and 5‐HT_7_ receptors, and as a partial agonist to 5‐HT_1A_ receptors, but it has low affinity for 5‐HT_2C_, H_1_ and M_1_ receptors.^25^, ^26^ Lurasidone has demonstrated efficacy and tolerability across multiple short‐term and long‐term clinical trials.^27^, ^28^, ^29^, ^30^, ^31^, ^32^, ^33^ The relatively weak affinity of lurasidone for H_1_ and M_1_ receptors may be responsible for its reported minimal effects on weight, lipids, and glycemic indices in short‐ and long‐term studies of lurasidone in the treatment of schizophrenia.^34^, ^35^, ^36^, ^37^, ^38^, ^39^, ^40^, ^41^, ^42^ These minimal effects on metabolic parameters are especially relevant given the high prevalence of metabolic syndrome among those with schizophrenia.^43^

Regarding the effects of lurasidone on anxiety symptoms, the receptor profile (antagonistic activity for 5‐HT_7_ receptors and partial agonistic activity for 5‐HT_1A_ receptors) and the data of animal models suggest the potential of lurasidone.^25^, ^26^ In addition, it has been reported that lurasidone improved the PANSS Marder 5 factor anxiety/depression scores.^44^, ^45^ Lurasidone has also been found to improve anxiety symptoms with patients with Bipolar I depression^46^, ^47^, ^48^; however, no studies on the efficacy of lurasidone for schizophrenia patients with comorbid anxiety symptoms have been reported.

The aim of the current study was to investigate whether lurasidone is effective in treating comorbid anxiety symptoms in patients with schizophrenia. In addition, the study aimed to determine whether lurasidone possesses anxiolytic properties in the absence of concomitant anxiolytic medication. To accomplish this, we pooled the data from five Phase 3 randomized trials and conducted subgroup analyses considering the severity of anxiety symptoms. For these analyses, we focused on the lurasidone treatment doses of 40 and 80 mg, which are the approved doses in Japan and are also considered to provide sufficient antipsychotic efficacy. Since lurasidone has been approved for a shorter period in Japan compared to North America, we considered it important to present data specifically for these doses.

## METHODS

Data for the current post hoc analyses were pooled from five multicenter, Phase 3 trials of lurasidone for acute schizophrenia: (1) ClinicalTrials.gov No. NCT00549718,^38^ (2) NCT00615433,^35^ (3) NCT00790192,^39^ (4) NCT01614899,^29^ and (5) EudraCT No. 2016‐000060‐42.^31^ The protocols of each trial were approved by the Ethics Committee at each participating center, and written informed consent was obtained from each patient following an explanation of study procedures in each trial.

### Design and participants

All five studies were randomized, double‐blind, placebo‐controlled trials of lurasidone for the treatment of acute schizophrenia. Only data from the lurasidone 40 and 80 mg/day dosage groups (and placebo) were included in the current report. Two studies included both 40 and 80 mg/day doses of lurasidone, while two studies included only the 40 mg/day dose of lurasidone, and one study included only the 80 mg/day dose of lurasidone. Excluded from the current analysis were higher doses of lurasidone (120, 160 mg/day) and comparator antipsychotics (olanzapine, quetiapine XR). Included participants in this post hoc analysis are summarized in Figure S1.

Similar inclusion and exclusion criteria were used for all five studies. In brief, all studies enrolled male and female patients 18–75 years of age who met the Diagnostic and Statistical Manual of Mental Disorders, fourth edition, text revision (DSM‐IV‐TR) criteria for schizophrenia.^49^ To be eligible, patients also were required to have an exacerbation of psychotic symptoms, with a PANSS total score of ≥80, including a score of ≥4 (moderate) on two or more of the following PANSS items: delusions, conceptual disorganization, hallucinations, suspiciousness, and unusual thought content at screening and baseline visits. Key exclusion criteria were diagnosis of schizoaffective disorder, an acute or unstable medical condition, alcohol or other drug abuse/dependence within the past 3–12 months; patients were also excluded who were judged by the study investigator to be at risk for suicide or injury to self or others. As‐needed treatment with anxiolytics and sedatives/hypnotics was permitted in each study.

### Assessments

Efficacy measures included the PANSS total and positive, negative, and general psychopathology subscale scores. Also, we evaluated anxiolytic response using the PANSS G2 anxiety item score. In addition, the PANSS Lindenmayer 5‐factor score was analyzed, which includes validated factors that measure outcomes not specifically evaluated by the PANSS subscale scores (notably, anxiety/depression).^50^ Finally, the Montgomery‐Åsberg Depression Rating Scale (MADRS)^51^ and the Calgary Depression Scale for Schizophrenia score (CDSS)^52^ were used to assess depressive symptoms.

Safety endpoints included assessment of adverse events (AEs) measured at each visit and laboratory tests measured at baseline and Week 6. In addition, discontinuation rates and reasons for discontinuation were examined.

### Statistical analysis

The efficacy analysis population included all randomized patients who received at least one dose of study drug and had both baseline and at least one post‐baseline assessment on the PANSS. All efficacy analyses were performed on two baseline anxiety subgroups, a “severe” anxiety subgroup (PANSS G2 anxiety item score ≥ 4 [moderate or more severe anxiety]) and a non‐severe anxiety subgroup (PANSS G2 score < 4 [mild or less than mild anxiety]).

Efficacy measures derived from the PANSS were analyzed using a mixed model for repeated measures (MMRM), with fixed factors of pooled study center, visit as a categorical variable, treatment and treatment‐by‐visit interactions, and baseline PANSS score as a covariate. The model included a specific contrast to evaluate the estimated change from baseline to Week 6. An unstructured covariance matrix was used for the within‐subject correlation, and Kenward–Roger's approximation was used to calculate the denominator degree of freedom. Changes from baseline to Week 6 in the MADRS total score and CDSS total score were examined using analysis of covariance (with baseline score as covariate). For anxiety, clinical response was defined as a PANSS G2 anxiety item score of <4 at Week 6 (last observation carried forward, LOCF) in the severe anxiety group (baseline PANSS G2 score ≥ 4). This means that moderate or more severe anxiety symptoms were improved to mild or less than mild anxiety symptoms. The proportion of responders in each treatment group at Week 6 (LOCF) was examined descriptively. As an additional analysis, in the subgroup with severe anxiety, Pearson correlation coefficients were calculated between the PANSS G2 anxiety score and the PANSS positive subscale score.

The safety analysis included all patients who received at least one dose of study medication. Safety analyses included descriptive reporting of change from baseline in laboratory parameters, discontinuation rates, and reasons for discontinuation. A subgroup analysis of safety outcomes was performed for the severe (vs. non‐severe) anxiety subgroups. The difference in safety outcomes was also evaluated based on whether or not patients received any concomitant anxiolytics during the study.

## RESULTS

### Subject disposition and baseline characteristics

A total of 1788 patients were included in the pooled study population that consisted of patients randomized to 40 or 80 mg of lurasidone, or placebo, and received at least one dose of study medication (safety analysis population). Of these, 878 (49.1%) were in the severe anxiety group and 910 (50.9%) were in the non‐severe anxiety group. Target patients were shown in Figure S1.

The severe anxiety group was similar to the non‐severe anxiety group in terms of mean age, sex ratio, duration of schizophrenia, PANSS positive and negative subscale scores (Table 1). Compared to the non‐severe anxiety group, the severe anxiety group had higher PANSS G2‐anxiety item scores (4.5 vs. 2.6 in placebo), were more likely to have had ≥4 previous hospitalization for schizophrenia (60.1% vs. 50.2%), and had slightly higher PANSS total scores (mean, 102.5 vs. 96.4) and slightly higher prior use of anxiolytic medication (56.2% vs. 51.7%). Use of concomitant anxiolytics medication during the study was similar in the severe (57.7%) and non‐severe (55.0%) anxiety groups. Prior or concomitant use of anxiolytics in each treatment group is shown in Table S1.

**Table 1:** Baseline characteristics (efficacy analysis set).

	Severe anxiety			Non‐severe anxiety		
	Placebo (*N* = 356)	Lurasidone		Placebo (*N* = 377)	Lurasidone	
		40 mg (*N* = 334)	80 mg (*N* = 175)		40 mg (*N* = 297)	80 mg (*N* = 221)
Female, %	41.3	44.0	38.3	34.0	37.0	35.3
Age, mean years	39.4	40.2	39.8	38.9	40.9	39.8
Race, %						
White	56.2	55.7	44.0	40.6	32.7	26.2
Black or African American	13.2	12.6	16.0	14.3	15.5	14.9
Asian	28.9	30.2	39.4	42.2	50.5	57.0
Duration of disease, mean years	12.8 (*n* = 320)	12.3 (*n* = 284)	13.4	12.4 (*n* = 345)	14.0 (*n* = 266)	13.5
≥4 previous hospitalizations, %	58.6 (*n* = 292)	61.4 (*n* = 272)	61.1 (*n* = 113)	50.5 (*n* = 299)	47.7 (*n* = 214)	53.4 (*n* = 131)
Baseline scores, mean						
PANSS G2 (anxiety)	4.5	4.5	4.3	2.6	2.6	2.6
PANSS total	102.3	103.6	101.0	96.0	96.9	96.4
PANSS positive subscale	26.1	26.3	25.8	25.2	25.3	25.7
PANSS negative subscale	24.2	24.7	24.6	24.5	24.7	24.6
PANSS general psychopathology subscale	52.1	52.6	50.5	46.3	46.8	46.0

Abbreviation: PANSS, Positive and Negative Syndrome Scale.

Overall discontinuation rates at 6 weeks were generally similar for the severe anxiety and non‐severe anxiety groups (30.8% vs. 28.4%). Discontinuation rates due to lack of efficacy were similar for the severe and non‐severe anxiety groups. However, discontinuation due to AEs was higher in patients treated with lurasidone 80 mg in the severe versus non‐severe anxiety group (8.4% vs. 1.4%). The subjects' disposition is shown in Table S2.

### Efficacy results for anxiety symptoms

#### Severe anxiety group

**Figure 1:**
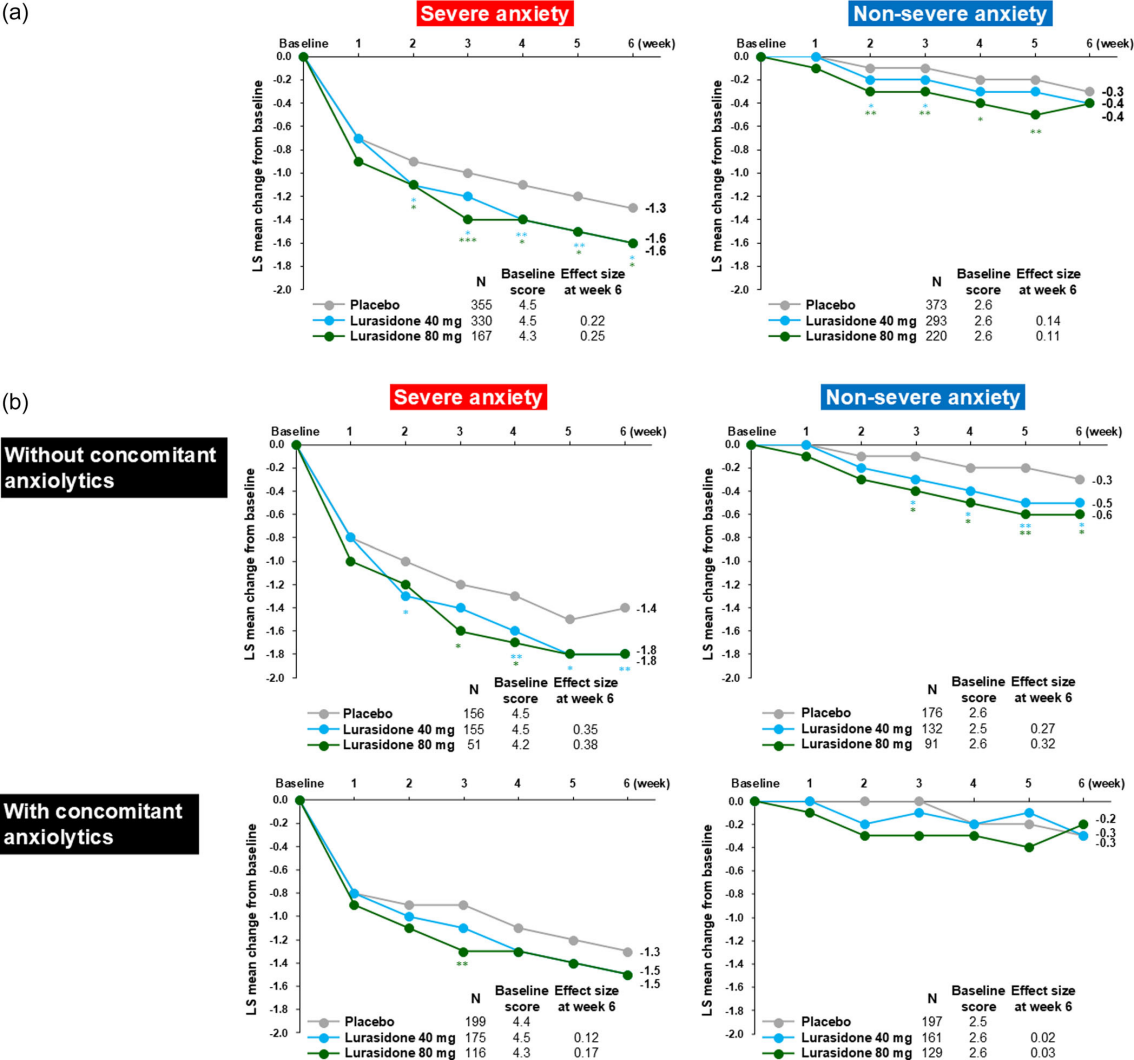
Change from baseline in Positive and Negative Syndrome Scale (PANSS) anxiety item score (G2) (mixed model for repeated measures [MMRM]). (a) Total sample (efficacy analysis population). (b) Subgroup with or without concomitant anxiolytics. LS, least square. *p < 0.05, **p < 0.01, and ***p < 0.001.

Treatment with lurasidone 40 and 80 mg was associated with a significant reduction in the PANSS G2 anxiety score, with an onset of significant improvement at Week 2 that was sustained to Week 6 endpoint (Figure 1a). The proportion of responders (PANSS G2 score < 4) at Week 6 (LOCF) was higher than placebo (50.3%) for lurasidone 40 mg (60.7%; number needed to treat [NNT]: 10 [95% confidence interval (CI): 5–34]), and 80 mg (59.8%; NNT: 11 [95% CI: 5–185]). In the subgroup of patients that were not receiving concomitant anxiolytics (Figure 1b), treatment with lurasidone was associated with a significant reduction in the PANSS G2 anxiety score on both the 40 mg dose (Week 6 effect size, 0.35) and the 80 mg dose (Week 6 effect size, 0.38). In the subgroup of patients that were receiving concomitant anxiolytics (Figure 1b), treatment with lurasidone was associated with somewhat reduced endpoint improvement in the mean PANSS G2 anxiety score compared to the subgroup without concomitant anxiolytics on both the 40 mg dose (−1.5 vs. −1.8; effect size 0.12 vs. 0.35) and the 80 mg dose (−1.5 vs. −1.8; effect size 0.17 vs. 0.38).

#### Non‐severe anxiety group

In the non‐severe anxiety group (efficacy population), with low baseline G2 anxiety scores, modest improvement was observed during treatment with lurasidone 40 and 80 mg that was not significant at Week 6 (Figure 1a). In the subgroup of patients that were not receiving concomitant anxiolytic medication (Figure 1b), treatment with lurasidone was associated with a significant reduction in the PANSS G2 anxiety score on both the 40 mg dose (effect size, 0.27) and the 80 mg dose (effect size, 0.32). In the subgroup of patients that were receiving concomitant anxiolytic medication (Figure 1b), the baseline PANSS G2 anxiety score was low, and no lurasidone treatment effect (vs. placebo) was observed.

### Efficacy results for overall symptoms

#### Severe anxiety group

**Figure 2:**
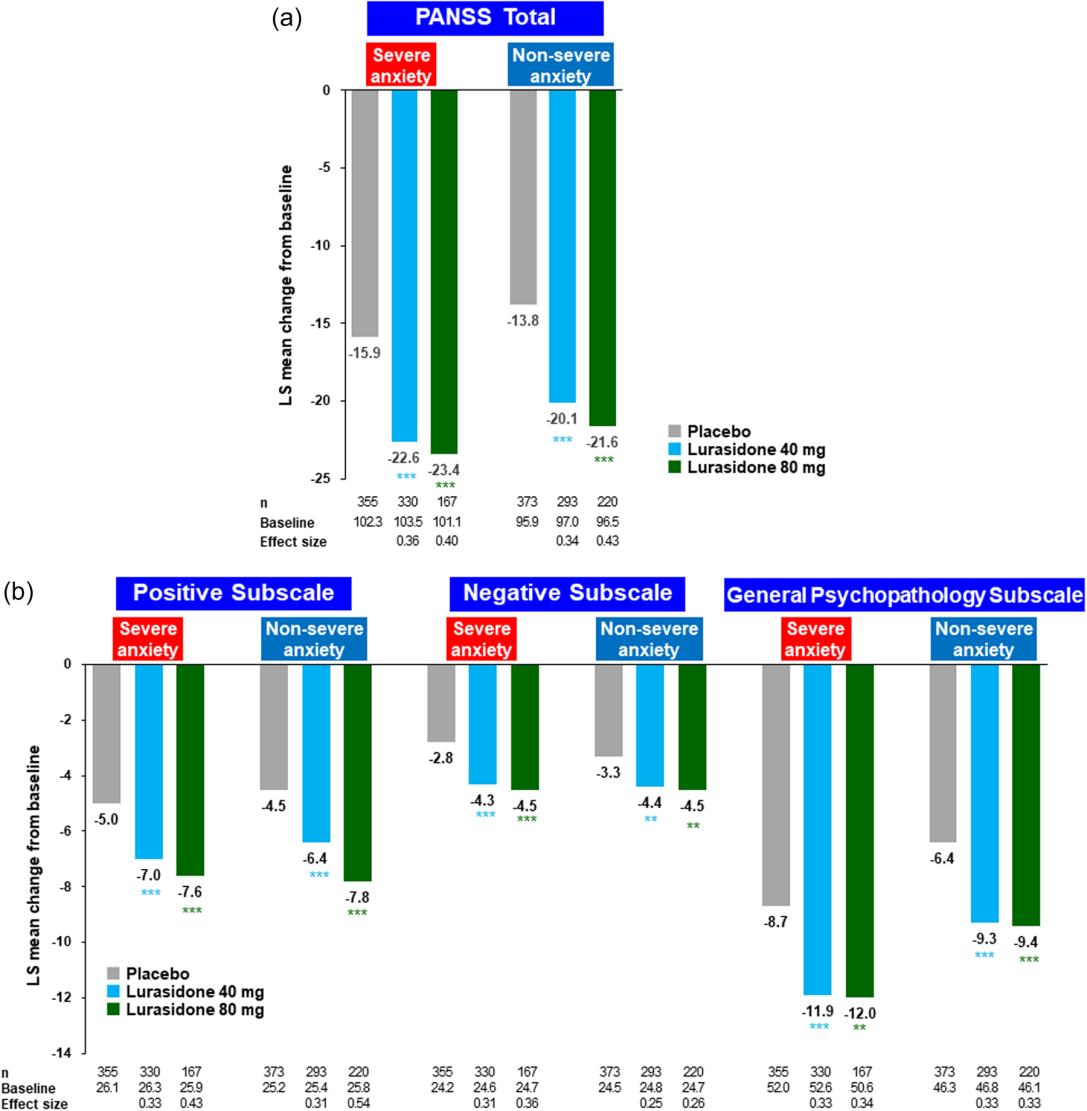
Change from baseline in Positive and Negative Syndrome Scale (PANSS) total and subscale scores at Week 6 (mixed model for repeated measures [MMRM], efficacy analysis set). (a) PANSS total score. (b) PANSS subscale scores. LS, least square. *p < 0.05, **p < 0.01, and ***p < 0.001.

Treatment with lurasidone 40 and 80 mg was associated with a significant endpoint reduction in the PANSS total score (Figure 2a), and in the PANSS positive, negative, and general psychopathology subscale scores (Figure 2b). A similar magnitude of significant endpoint improvement was observed on the PANSS Lindenmayer 5‐factor scores (negative symptoms, excitement, cognitive disorders, positive symptoms, and anxiety/depression; Table 2). The only dose–response effect that was observed was a larger endpoint effect size for lurasidone 80 mg versus 40 mg on the PANSS positive symptom subscale (effect size, 0.43 vs. 0.33) and the Lindenmayer positive symptom factor (effect size, 0.41 vs. 0.31).

**Table 2:** Change from baseline in Positive and Negative Syndrome Scale (PANSS) Lindenmayer 5‐factor model score at Week 6 (efficacy analysis set).

PANSS Lindenmayer 5‐factor model (MMRM)		Severe anxiety			Non‐severe anxiety		
		Placebo (*N* = 355)	Lurasidone		Placebo (*N* = 373)	Lurasidone	
			40 mg (*N* = 330)	80 mg (*N* = 167)		40 mg (*N* = 293)	80 mg (*N* = 220)
Negative symptoms	Baseline, mean	20.8	21.3	21.3	20.8	21.0	20.6
	LS mean change	−3.0	−4.3	−4.3	−3.0	−4.2	−4.1
	*p*‐Value (vs. placebo)		<0.001	0.003		0.001	0.004
	Effect size		0.31	0.32		0.28	0.27
Excitement	Baseline, mean	12.7	12.4	12.6	10.4	10.8	11.0
	LS mean change	−2.2	−3.2	−3.3	−1.5	−2.1	−2.5
	*p*‐Value (vs. placebo)		<0.001	0.003		0.018	<0.001
	Effect size		0.30	0.33		0.21	0.33
Cognitive disorders	Baseline, mean	16.1	16.2	15.5	16.0	16.3	16.4
	LS mean change	−2.2	−3.0	−3.2	−2.2	−3.1	−3.6
	*p*‐Value (vs. placebo)		0.001	0.001		<0.001	<0.001
	Effect size		0.27	0.34		0.30	0.48
Positive symptoms	Baseline, mean	15.7	15.9	15.5	15.7	15.6	15.6
	LS mean change	−3.1	−4.2	−4.5	−3.1	−4.0	−4.6
	*p*‐Value (vs. placebo)		<0.001	<0.001		0.005	<0.001
	Effect size		0.31	0.41		0.24	0.40
Anxiety/depression	Baseline, mean	17.1	17.3	16.3	13.2	13.0	12.8
	LS mean change	−3.7	−4.6	−4.6	−2.3	−2.9	−2.6
	*p*‐Value (vs. placebo)		0.002	0.012		0.017	0.228
	Effect size		0.27	0.27		0.21	0.12

Abbreviations: LS, least square; MMRM, mixed model for repeated measures.

In the severe anxiety subgroup, Pearson correlation coefficients between the PANSS G2 anxiety and positive subscale scores at baseline were 0.261 for the 40 mg group and 0.014 for the 80 mg group, indicating weak or no correlation. For baseline‐to‐Week‐6 change scores, Pearson correlation coefficients were 0.405 for the 40 mg group and 0.431 for the 80 mg group, indicating a moderate level of correlation.

#### Non‐severe anxiety group

Treatment with lurasidone 40 and 80 mg was associated with a significant endpoint reduction in the PANSS total score (Figure 2a), and in the PANSS positive, negative, and general psychopathology subscale scores (Figure 2b). A similar magnitude of significant endpoint improvement was observed on the PANSS Lindenmayer 5‐factor model scores (negative symptoms, excitement, cognitive disorders, positive symptoms, and anxiety/depression; Table 2). For this non‐severe anxiety subgroup, a dose–response effect was observed on both the positive subscale on the PANSS (Figure 2b) and the positive symptom and cognitive disorders factors on the Lindenmayer 5‐factor model.

Effect sizes for lurasidone versus placebo were generally similar for the severe anxiety group and the non‐severe anxiety group on the other PANSS subscales and Lindenmayer 5 factors.

### Efficacy results for the depressive symptoms

**Figure 3:**
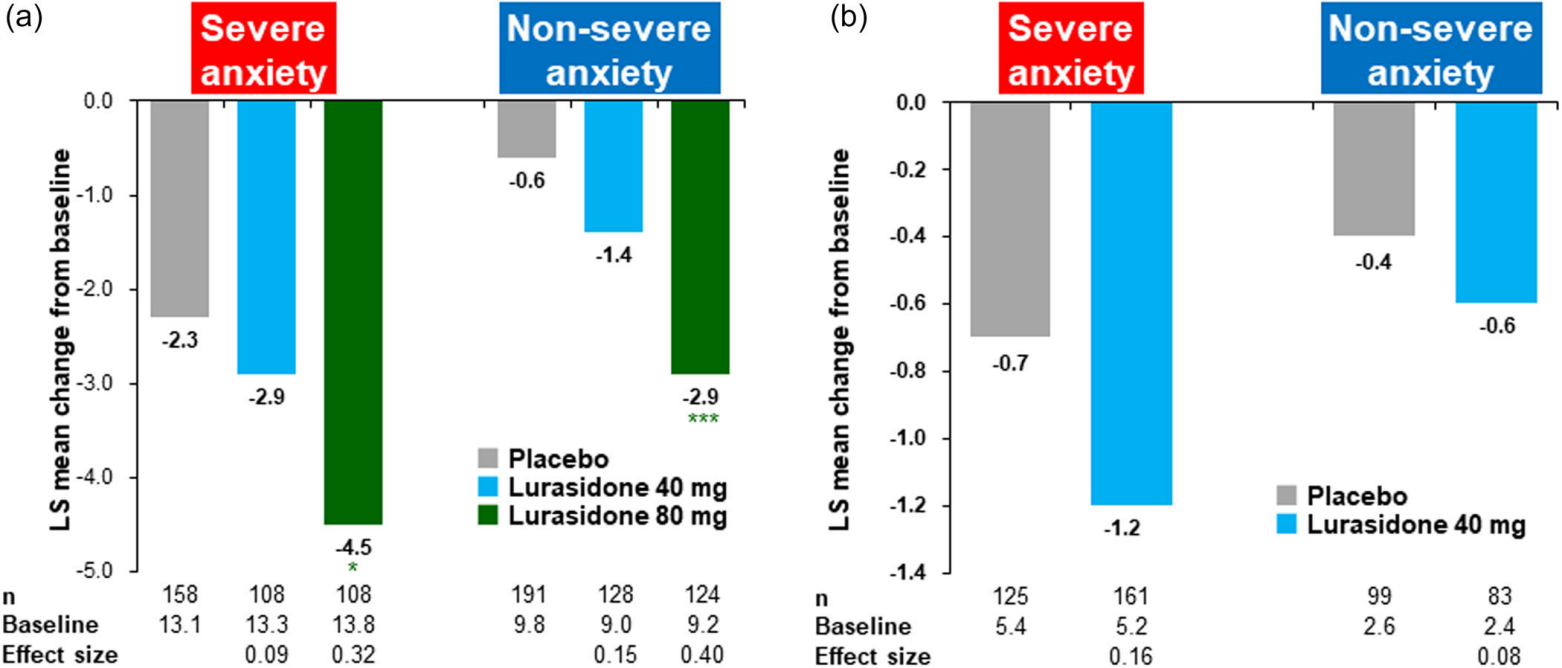
Change from baseline to 6 weeks (last observation carried forward [LOCF]) in Montgomery‐Åsberg Depression Rating Scale score (MADRS) total and Calgary Depression Scale for Schizophrenia score (CDSS) total score (analysis of covariance [ancova], efficacy analysis population). (a) MADRS. (b) CDSS. LS, least square. *p < 0.05, ***p < 0.001.

In both the severe and non‐severe anxiety groups, the MADRS total score was significantly decreased with 80 mg of lurasidone compared to placebo at Week 6 (Figure 3a). In the 40 mg lurasidone group, a nonsignificant reduction in MADRS total score was evident in both the severe and non‐severe anxiety groups. A dose–response effect was observed for endpoint change in the MADRS, with larger effect sizes for lurasidone 80 mg versus 40 mg in both the severe anxiety subgroup (0.32 vs. 0.09) and the non‐severe anxiety subgroup (0.40 vs. 0.15).

Only one of the five original trials used the CDSS as a depression outcome measure, and this study only investigated the 40 mg dose of lurasidone. Both the severe and non‐severe anxiety groups showed a greater reduction in CDSS total score with 40 mg of lurasidone than with placebo at Week 6, but the differences were not significant (Figure 3b).

### Safety results

There was no difference in the proportion of all AEs between lurasidone and placebo in either the severe or non‐severe anxiety groups (Table 3). The most common AE was headache, but there was no difference between lurasidone and placebo in both the severe anxiety group and the non‐severe anxiety group on this AE. The next most common AE was akathisia, which was more common with lurasidone than with placebo in both the severe and non‐severe anxiety groups. Furthermore, in both the severe and non‐severe anxiety groups, there was a tendency for the proportion to be higher at 80 mg than at 40 mg. In addition, nausea and somnolence were higher with lurasidone than with placebo in both the severe and non‐severe anxiety groups. There were no significant differences in the proportion of other AEs between placebo and lurasidone.

**Table 3:** Adverse events (safety analysis set).

	Severe anxiety			Non‐severe anxiety		
	Placebo (*N* = 364)	Lurasidone		Placebo (*N* = 385)	Lurasidone	
		40 mg (*N* = 336)	80 mg (*N* = 178)		40 mg (*N* = 304)	80 mg (*N* = 221)
Any AEs, *n* (%)	251 (69.0%)	214 (63.7%)	129 (72.5%)	214 (55.6%)	195 (64.1%)	140 (63.3%)
Common AEs,^a^ *n* (%)						
Headache	43 (11.8%)	44 (13.1%)	21 (11.8%)	39 (10.1%)	34 (11.2%)	13 (5.9%)
Akathisia	10 (2.7%)	30 (8.9%)	21 (11.8%)	5 (1.3%)	19 (6.3%)	25 (11.3%)
Insomnia	35 (9.6%)	28 (8.3%)	14 (7.9%)	31 (8.1%)	20 (6.6%)	19 (8.6%)
Nausea	20 (5.5%)	22 (6.5%)	19 (10.7%)	11 (2.9%)	18 (5.9%)	11 (5.0%)
Somnolence	8 (2.2%)	20 (6.0%)	12 (6.7%)	6 (1.6%)	21 (6.9%)	9 (4.1%)
Anxiety	22 (6.0%)	20 (6.0%)	16 (9.0%)	23 (6.0%)	17 (5.6%)	8 (3.6%)

Abbreviation: AE, adverse event.

^a^ In ≥5% of patients in total lurasidone.

There were no clinically meaningful differences between lurasidone and placebo in both the severe and non‐severe anxiety groups on any of the laboratory parameters (Table S3).

## DISCUSSION

The results of this post hoc analysis suggest that lurasidone may improve anxiety symptoms in schizophrenia patients who have more severe anxiety symptoms. In the non‐severe anxiety symptom group, lurasidone did not significantly improve anxiety symptoms at Week 6 (though significant effects were evident at Weeks 2–5). However, the mean baseline PANSS G2 (anxiety) score in this group was relatively low, making it an anxiolytic treatment effect difficult to observe due to a floor effect.

The mechanism for the anxiolytic effects of lurasidone is not fully known. Lurasidone has been reported to act as a 5‐HT_1A_ receptor partial agonist and 5‐HT_7_ receptor antagonist.^25^, ^26^ There is strong evidence for the anxiolytic effects of 5‐HT_1A_ agonists and some evidence for anxiolytic effects of 5‐HT_7_ antagonists in animal studies.^53^, ^54^ Consistent with this literature, lurasidone has been found to improve anxiety‐like symptoms in animal models.^25^ Thus, the pharmacological profile of lurasidone may contribute to the anxiolytic effects observed in this study. It is also possible that the anxiolytic effects of lurasidone in this patient population might be an indirect effect that is secondary either to a sedative effect of the drug or to improvement in the positive symptoms. In the pooled studies in this analysis, “somnolence” was the preferred term in MedDRA (Medical Dictionary for Regulatory Activities) used to code for all sedative‐related AEs (e.g., sleepiness, drowsiness). These effects occurred infrequently in the severe anxiety subgroup, with rates of 6.0%/6.7% (lurasidone 40/80 mg); therefore, it seems unlikely that the anxiolytic effect observed in the current study could be attributable to so small a patient subgroup.

A correlational analysis of baseline‐to‐Week‐6 change scores found a moderate level of correlation (0.40–0.43) between the PANSS G2 anxiety and positive subscale scores, indicating that some of the anxiolytic effect of lurasidone in this schizophrenic population was attributable to the antipsychotic effect of the drug, but that improvement in anxiety symptoms on lurasidone may also be attributable to a direct anxiolytic effect. A previously reported correlational analysis of Week 6 PANSS factor change scores found a Pearson correlation coefficient score of 0.52 between the Marder PANSS positive symptom and anxiety/depression factor change scores (similar to the current study). When this cross‐correlation was corrected for, lurasidone was found to have anxiolytic effect.^55^ This report and the current results indicate a potential dual anxiolytic effect for lurasidone, both direct and indirect.

The current results indicate that anxiety symptoms improve with lurasidone treatment even without the addition of concomitant anxiolytics. In fact, the Week 6 effect sizes for lurasidone were slightly higher in the subgroup of patients who did not receive concomitant benzodiazepine medication. Taken together, these findings suggest that lurasidone may have a direct effect on anxiety levels in this clinical population and that use of benzodiazepines could be reduced. Given the potential for misuse and dependence,^56^ and the reported association of higher mortality rates with long‐term benzodiazepine use among individuals with schizophrenia,^17^ reductions in the use of benzodiazepines for patients with schizophrenia might be an important clinical goal. Further research, however, is needed to confirm whether benzodiazepines use can be reduced when lurasidone treatment of schizophrenia is initiated.

The potential anxiolytic effects of lurasidone may carry over to other outcomes. It has been reported that anxiety symptoms in schizophrenia are associated with impaired social functioning,^8^ decreased quality of life,^8^, ^9^ and suicidality.^4^, ^5^, ^6^, ^57^ Anxiety symptoms have also been reported to be one of the early signs of psychotic relapse.^3^, ^58^ Improving anxiety symptoms may therefore lead to improved social functioning and quality of life, reduced risk of suicide, and possible reduction in relapse risk.

Some evidence exists regarding the effectiveness of other atypical antipsychotics for schizophrenia patients with anxiety symptoms. Aripiprazole has been shown to reduce social anxiety symptoms in patients with social anxiety symptoms.^18^ Olanzapine has been reported to improve anxiety symptoms in patients with a PANSS G2 score of 3 or higher.^19^ These were open‐label, small *N*, single‐arm studies, so there is limited evidence of the anxiolytic effects of other atypical antipsychotics on schizophrenia patients with anxiety symptoms. In contrast, in addition to the data presented in this report, lurasidone has been found to have greater efficacy in the short‐term treatment of schizophrenia than placebo in reducing the PANSS 5‐factor anxiety/depression factor score in one pooled study dataset and one 6‐week study.^31^, ^45^ Because each atypical antipsychotic has a different pharmacological profile, it would be of interest to investigate how different atypical antipsychotics have different effects on anxiety symptoms in head‐to‐head studies.

In addition to effects on anxiety symptoms, the present results suggest lurasidone may improve other prominent symptoms in schizophrenia regardless of the severity of anxiety symptoms. In particular, the current results indicate that 80 mg of lurasidone may improve depression symptoms (assessed by MADRS), regardless of the severity of anxiety symptoms. However, considering that among the five studies included in this study, three studies used the MADRS, and only one study used CDSS (and in that study, only 40 mg of lurasidone was used), further investigation is therefore needed to determine which dose of lurasidone has this potential antidepressant effect. The current results also suggest that lurasidone improves overall psychiatric symptoms, regardless of the severity of anxiety symptoms. In the subscale analysis, the PANSS positive subscale showed 80 mg of lurasidone was more effective than 40 mg, regardless of the severity of anxiety symptoms. For both the PANSS Negative subscale and PANSS general psychopathology subscale, 40 and 80 mg of lurasidone had similar effect sizes in comparison to placebo. From these results, it appears that lurasidone has a dose‐dependent effect on positive symptoms, but that 40 and 80 mg may have similar effects on other symptoms.

Results of the analysis of the PANSS Lindenmayer 5‐factor model also showed that both the 40 and 80 mg doses of lurasidone were effective for all factor scores except for the anxiety/depression factor score, regardless of the severity of anxiety symptoms. For the Lindenmayer anxiety/depression factor score, both 40 and 80 mg in the severe anxiety group and 40 mg in the non‐severe anxiety group showed a significant effect compared to placebo at Week 6, but the effect for 80 mg in the non‐severe anxiety group was not significant. This may be due to the low baseline score on this factor in the non‐severe anxiety group, similar to what was observed with the PANSS G2 score.

In the severe anxiety group, the high rate of responders in the placebo group deserves comment (50.3% vs. 60.7% and 59.8% for lurasidone 40 and 80 mg, respectively). This appears to be attributable to our a priori decision to use the baseline criterion for “severe” anxiety as the threshold criterion for declaring a patient to be a “responder.” As a consequence, a patient could achieve responder status with only a very modest reduction (<1‐point) in their PANSS G2 anxiety severity score.

Regarding overall AEs, there was no meaningful difference between the severe anxiety group and the non‐severe anxiety group for both the 40 and 80 mg doses, but there was a tendency for the 80 mg dose to have more AEs within the severe anxiety group. Akathisia was more common and occurred at a higher rate than the placebo in the severe anxiety group. This was consistent with the results of the original individual studies that were pooled here.^29^, ^31^, ^35^, ^38^, ^39^ There were no other major differences in the tendency for AEs to occur between the severe anxiety group and the non‐severe anxiety group. Similarly, no clinically meaningful changes were observed in either the severe anxiety group or the non‐severe anxiety group in metabolic parameters. No new concerns were found regarding AEs compared to the original studies.

The results of this study need to be put in the context of several limitations. First, the study was a post hoc analysis of pooled data across five studies and thus should be considered to be exploratory. A prospective study confirming the current findings is indicated with appropriate adjustments for multiple comparisons. Alternatively, instead of pooling data, a meta‐analysis of individual patient data may have provided additional insight. Second, patients included in the studies pooled here met specific inclusion and exclusion criteria. Generalization of the findings to a broader range of patients is uncertain. Third, treatment was 6 weeks in duration in all studies. The longer term anxiolytic effects of lurasidone should also be investigated. Fourth, a single PANSS item (G2) was used to assess the severity of anxiety symptoms. Additional prospective treatment research is needed utilizing reliable and validated anxiety symptom scales. Fourth, the absence of a comparator antipsychotic in the current analysis, as well as the lack of RCTs that examine the anxiolytic efficacy of other antipsychotic medication in patients with a diagnosis of schizophrenia, means that we cannot determine whether the anxiolytic efficacy reported here for lurasidone is unique to its pharmacological profile or is a more general effect of antipsychotic therapy. Finally, it is important to note that the five clinical trials that were pooled in this post hoc analysis required patients to meet DSM‐IV‐TR criteria for schizophrenia as the primary diagnosis. The severity of concurrent anxiety as a symptom was recorded at each assessment visit, but the presence of a comorbid anxiety as a DSM‐IV‐TR diagnosis was not systematically recorded. This is perhaps unfortunate. We know that both symptomatic and syndromic levels of anxiety are associated with greater functional impairment, higher risk of suicidality, and overall poorer outcomes. However, disentangling “causal” effects is complex and beyond the scope of the current data.

In conclusion, the results suggest that treatment with 40 or 80 mg/day of lurasidone, compared to placebo, improves anxiety symptoms in schizophrenia patients with moderate to severe anxiety, at least in part, through a direct effect. Furthermore, this effect was observed in patients receiving lurasidone monotherapy, without concomitant anxiolytics, suggesting that the anxiolytic effects are more directly due to lurasidone treatment. In addition, lurasidone administration improved overall symptoms, including depressive symptoms, positive symptoms, and negative symptoms, regardless of the severity of anxiety symptoms. To achieve recovery, attention to anxiety symptoms in schizophrenia is increasingly essential, and these results suggest that lurasidone is a promising treatment option when the clinical presentation of schizophrenia is complicated by comorbid anxiety.

## AUTHOR CONTRIBUTIONS

**Takahiro Nemoto**: Conceptualization; methodology; supervision; writing—original draft; writing—review and editing. **Miyuki Okumura**: Conceptualization; methodology; writing—original draft; writing—review and editing. **Hidenori Maruyama**: Conceptualization; methodology; project administration; visualization; writing—original draft; writing—review and editing.

## CONFLICT OF INTEREST STATEMENT

Takahiro Nemoto belongs to the Department of Psychiatry and Implementation Science, Toho University Faculty of Medicine, which is funded by Nippon Life Insurance Company, and has received grants from Eisai Co., Ltd., Otsuka Pharmaceutical Co., Ltd., PDRadiopharma Inc., Shionogi & Co., Ltd., and Sumitomo Pharma Co., Ltd. as well as personal fees from Eisai Co., Ltd., Janssen Pharmaceuticals Inc., Lundbeck Japan, Meiji Seika Pharma Co., Ltd., Mitsubishi Tanabe Pharma Corporation, Nippon Boehringer Ingelheim Co., Ltd., Otsuka Pharmaceutical Co., Ltd., PDRadiopharma Inc., Sumitomo Pharma Co., Ltd., and Takeda Pharmaceutical Company Limited. Miyuki Okumura and Hidenori Maruyama are employed by Sumitomo Pharma Co., Ltd.

## ETHICS APPROVAL STATEMENT

The protocols of each of the five Phase 3 trials in this post hoc analysis were approved by the Ethics Committee at each participating center.

## PATIENT CONSENT STATEMENT

Written informed consent was obtained from each patient following an explanation of study procedures in each trial.

## CLINICAL TRIAL REGISTRATION

The registration for each of these five Phase 3 trials was (1) ClinicalTrials.gov No. NCT00549718 (Nasrallah et al.^38^), (2) NCT00615433 (Meltzer et al.^35^), (3) NCT00790192 (Loebel et al.^39^), (4) NCT01614899 (Higuchi et al.^29^), and (5) EudraCT No. 2016‐000060‐42 (Iyo et al.^31^).

## Supporting information

Supporting Information.

## Data Availability

The data from this study have not been made publicly available because the disclosure of individual data was not specified in the study protocol, and consent for public data sharing was not obtained from the participants.
