# Supplementary material for: Efficacy of lurasidone on anxiety symptoms in patients with schizophrenia: A pooled post hoc analysis of five randomized, placebo‐controlled trials
Source: PCN Rep. 2025 Nov 18;4(4):e70245. doi: 10.1002/pcn5.70245 (PMC12626723; doi:10.1002/pcn5.70245)

**Appendix A. Supplementary Figures and Tables**

**Supplementary Figure 1. Target patients**


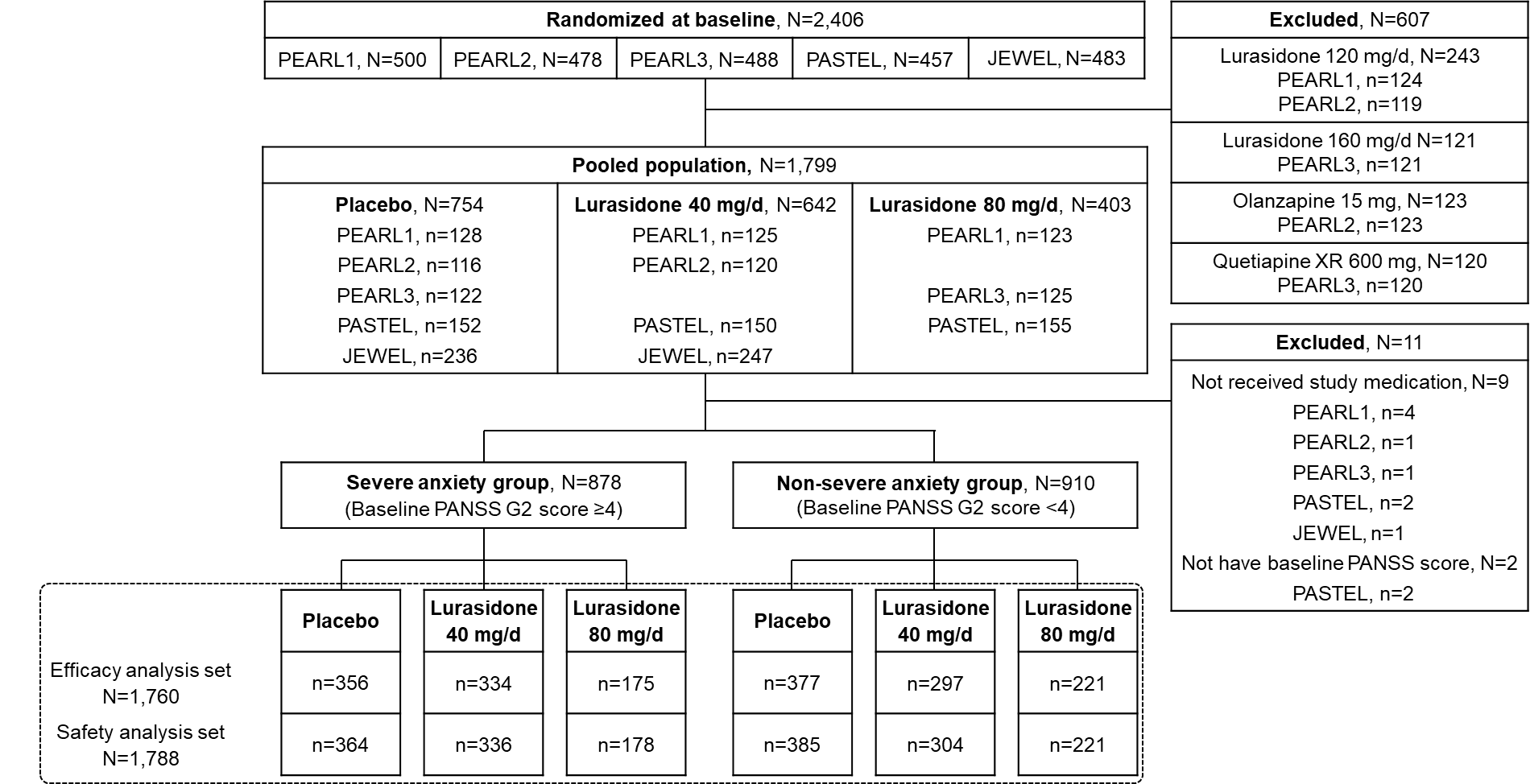


**Supplementary Table 1. Prior or concomitant use of anxiolytics (Efficacy analysis set)**


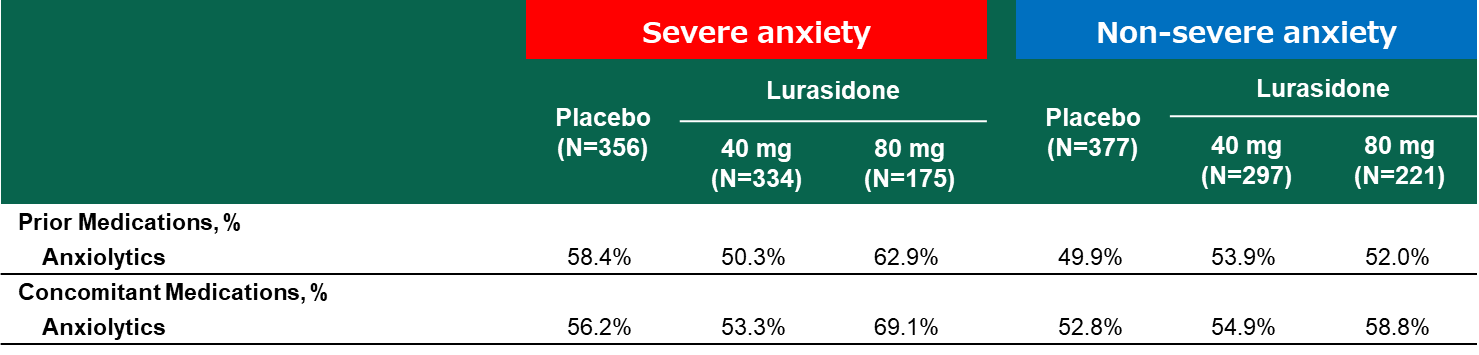


**Supplementary Table 2. Subjects disposition (Safety analysis population)**


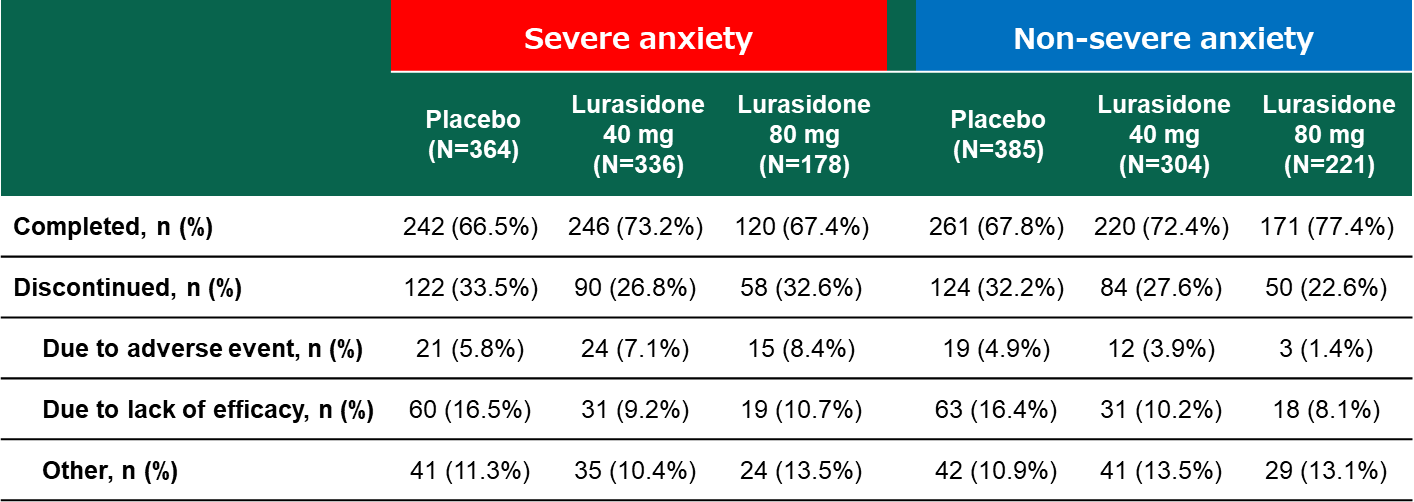


**Supplementary Table 3. Baseline and mean change to Week 6 (LOCF) in laboratory parameters and weight (Safety analysis set)**


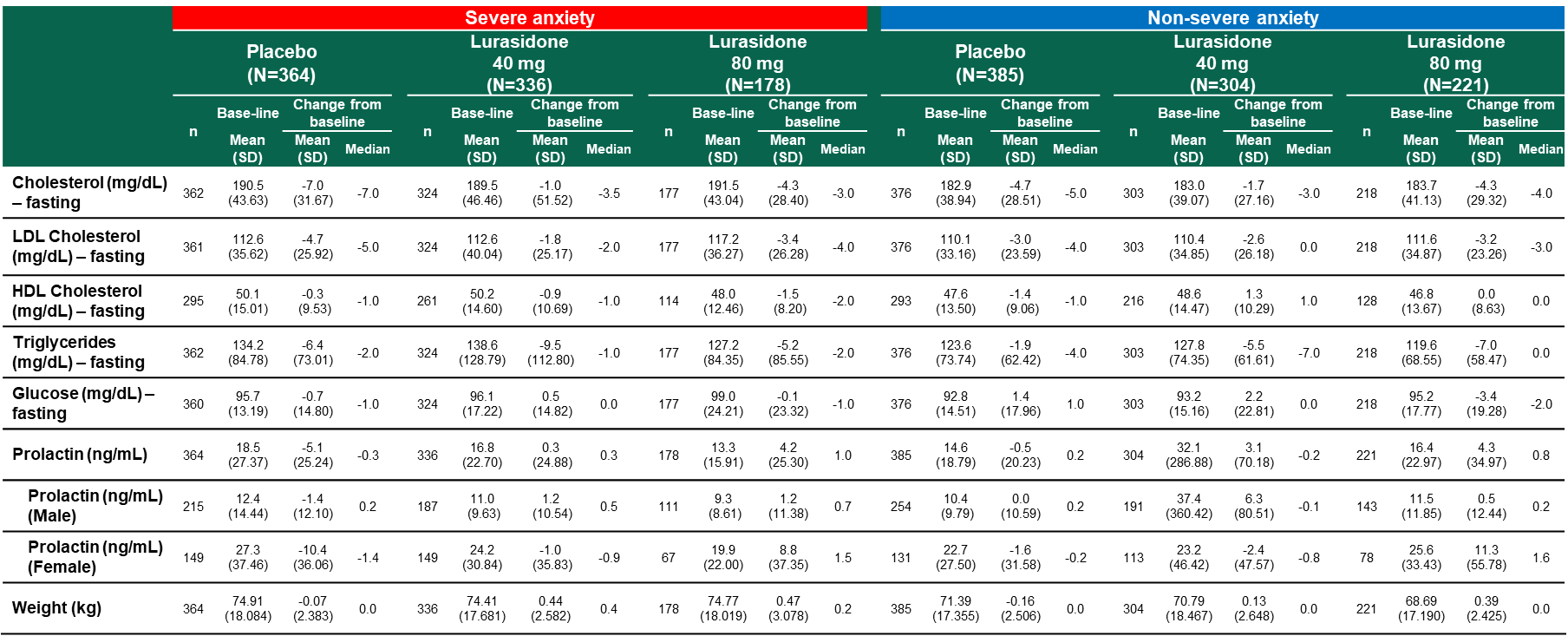

Supplement: Supplementary file 1 — Supporting Information. [file PCN5-4-e70245-s001.docx]
